# Phenotype and Genotype of Saudi Pediatric Patients With Neurofibromatosis Type 1: A Seven-Year Multicenter Experience From Saudi Arabia

**DOI:** 10.7759/cureus.37385

**Published:** 2023-04-10

**Authors:** Mohammed A Alfurayh, Nawaf K Alawad, Abdulaziz M Bin Akrish, Awad S Alharbi, Ahmed Sharahili, Abdulaziz S Bin Saleem, Muhammad T Alrifai

**Affiliations:** 1 Medicine and Surgery, King Saud Bin Abdulaziz University for Health Sciences College of Medicine, Riyadh, SAU; 2 College of Medicine, King Saud Bin Abdulaziz University for Health Sciences College of Medicine, Riyadh, SAU; 3 College of Medicine, King Abdulaziz Medical City, Riyadh, SAU; 4 Pediatrics, King Abdulaziz Medical City, Riyadh, SAU

**Keywords:** genetics, cafe-au-lait spots, cns, brain tumor, neurofibromatosis 1

## Abstract

Background

Neurofibromatosis type 1 (NF1) is a complex disorder. Genetics and environment might be attributed as the leading cause of NF1, which is characterized by multisystemic involvement. We aim to elaborate on Saudi children's NF1 phenotypes and genotypes.

Methods

This study was conducted in the Ministry of National Guard Health Affairs (MNGHA), Saudi Arabia including three tertiary hospitals, using a retrospective cohort method. Electronic charts were reviewed to extract the variables. All Saudi pediatric patients aged less than 18 with NF1 were included. Consecutive sampling was used due to the limited number of patients.

Results

The study included 160 patients (81 males) with an average age of 8.08 years.

Also, 33 (20.6%) patients had cutaneous neurofibroma while 31 (19.4%) patients had plexiform neurofibromas. Iris lisch nodules were seen in 33.75%. Optic pathway glioma was seen in 29 (18%) cases while non-optic pathway glioma was seen in 27 (17%) cases. Skeletal abnormalities were seen in 27 (17%) of cases. A first-degree relative with NF1 was seen in 83 (52%) of cases. Epilepsy was the presenting feature of 27 (17%) cases. Cognitive impairment was found in 15 (9.4%) patients. Genetic mutation was seen in 82/100 cases, the rest were negative. The types of mutations were as follows: nonsense 30 (36.6%); missense 20 (24.4%); splicing site mutation 12 (14.6%); frameshift 10 (12.2%); microdeletion 7 (8.5%); and whole gene deletion 3 (3.75%) patients. No phenotype-genotype correlation was seen.

Conclusion

In this cohort of Saudi pediatric patients with NF1, optic pathway glioma and other brain tumors were prevalent. The most common mutation is the nonsense mutation.

## Introduction

Neurofibromatosis (NF) is a complex hereditary disorder. The exact cause of NF is not known, yet environmental triggers and genetic predisposition might play a significant role in the pathogenesis of neurofibromatosis. Additionally, three heterogeneous disorders make up the definition of NF, which are neurofibromatosis type 1 (NF1), neurofibromatosis type 2 (NF2), and schwannomatosis [1]. All of these share the same principle of elevated risk of acquiring central nervous system (CNS) and peripheral nervous system (PNS) tumors [1]. NF1 is the most prevalent NF sub-type worldwide [2]. While NF1 is an autosomal-dominant (AD) inherited tumor syndrome, almost half of the time NF1 originates de novo in individuals with no known previous family history of NF1 [3].

Apart from nerve sheath tumors, NF1 puts affected children at risk of many other nervous system and non-nervous system problems such as long-bone dysplasia, autism, optic pathway glioma, and pheochromocytoma [4]. NF1 is a consequence of a germline mutation in the NF1 gene on chromosome number 17 [4]. On the other hand, NF2 originates from mutations in the NF2 gene on chromosome number 22 [5]. In contrast, schwannomatosis originates from mutations in either the integrase interactor 1 (INI1/SMARCB1) gene or the LZTR1 gene [6]. Probably other loci have not been discovered yet in chromosome number 22 [6].

Regarding the prevalence of NF1, a recent study that was done in Finland showed that the prevalence of NF1 among children is 1/4,088 individuals; nevertheless, the incidence rate of NF1 is ~1/2,000 live births [7]. Age-specified prevalence is highly important in NF1 patients where high mortality rates were observed at a young age and the prevalence declines as the age increases [7]. Moreover, a study demonstrated that symptoms of autism spectrum disorder (ASD) are more prevalent among males with NF1 when compared to females [8].

The pigmentary clinical manifestations of patients with NF1 are considered one of the most common clinical features [9]. Cafe-au-lait macules (CALMs) are flat, pigmentary skin lesions that are discovered during the early neonatal periods, and they constitute one entity of NF1 diagnostic criteria [9]. Furthermore, the number of CALMs at presentation is a significant predictor of the diagnosis and severity of NF1 [9]. Skinfold freckling may occur mainly in the axillary and inguinal regions, yet it can be found in other locations across the body [9]. Lisch nodules are melanocytic hamartomas of the eyes; they are seen in 95-100% of patients with NF1 [10]. Adding to these melanocytic abnormalities, children with NF1 are at a higher risk of other medical-related pathologies, one of which is dysplasia of the long bones [11]. Furthermore, a focal or general decrease in bone density was reported in people with NF1 increasing the bone fracture rates [11]. Children with NF1 can experience high blood pressure and other cardiac anomalies that are thought to be due to renal artery stenosis or cardiac valvular pathologies [12].

Optic and non-optic pathway gliomas were studied among patients with neurofibromatosis type 1. A study identified 24 (4.30%) patients out of 562 with non-optic gliomas and they were affected by NF1. Additionally, five patients out of the 24 were identified to have other CNS tumors in conjunction with the non-optic pathway gliomas (NOPGs) [13]. Moreover, NF patients with optic gliomas could present with either vision impairment or vision loss [13]. A study that was done in Germany showed that optic pathway gliomas (OPGs) were diagnosed in 134 out of 925 patients [14]. A study conducted in Israel showed that 57 out of 257 patients with NF1 were found to have optic pathway glioma [15]. Moreover, a study that included 306 patients who had done an MRI upon diagnosis showed that 45 patients had an OPG with the majority being asymptomatic [16]. A study done in Italy showed that 49 non-optic CNS tumors were observed in 35 pediatric patients, which is a relatively big number that needs to be considered when diagnosing a child with NF [17].

NF-associated phenotypes and genotypes have not been investigated thoroughly in Saudi Arabia. So, in this research, our main aim is to investigate the phenotype and genotype among pediatric patients with neurofibromatosis type 1 as well as to evaluate the possible correlation between a specific genotype and phenotype.

## Materials and methods

This is a retrospective study conducted at the neurology division of the pediatric department at King Abdullah Specialized Children’s Hospital (KASCH), King Abdulaziz Medical City (KAMC), and Ministry of National Guard Health Affairs (MNGHA), Riyadh, Saudi Arabia. This study was conducted using electronic medical records. The data of all Saudi children who have been diagnosed with neurofibromatosis type 1 were extracted from the MNGHA medical record system (BestCare 2.0A). BESTCare seeks to save the patient's medical history in an electronic medical record that begins with birth, in addition to patient interactive self-services that give a package of services following personal authentication through fingerprint, smart card, or medical record number. This study was conducted at three centers related to MNGHA in Saudi Arabia. The centers are King Abdulaziz Medical City (KAMC) - Riyadh, King Abdulaziz Medical City (KAMC) - Jeddah, and Prince Mohammed bin Abdulaziz Hospital (PMBAH) - Al Madinah. The inclusion criteria included all Saudi children aged less than 18 years who have been diagnosed with neurofibromatosis type 1 from January 2016 till September 2022. Patients with neurofibromatosis type 2 and other different variants were excluded. Due to the limited number of patients, all patients who met the inclusion criteria were selected through a non-probability consecutive sampling technique.

Statistical Package for Social Sciences (SPSS) software version 27.0.1 (IBM Corp., Armonk, NY) was used to analyze this study data. The sociodemographic characteristics' frequency, percentage, mean, and standard deviation of patients with neurofibromatosis type 1 were calculated. Data were analyzed in both descriptive and inferential statistics, including frequency and percentage for categorical variables, such as gender, co-morbidities, associated medical history, MRI findings, and the presence of diagnostic elements of NF type 1 such as café au lait spots, cutaneous or plexiform neurofibroma, Lisch nodules, skinfold freckling, skeletal involvement, first-degree relative with NF and genetic-related parameters, data on optic and non-optic pathway gliomas were obtained as well. In addition, numerical variables, such as the age of the patient and body mass index, were presented as mean +/- standard deviation. Inferential statistics were done to find associations and correlations between different variables, which included the type of genetic mutations and the clinical manifestation of NF1 patients. The student’s t-test and χ2 test were used to compare means and proportions, respectively. There is a statistically significant association between tested variables if p < 0.05.

This study was approved by the institutional review board at King Abdullah International Medical Research Center (KAIMRC), and the identifying number of the IRB approval was IRB/2170/22.

## Results

This study included 160 Saudi pediatric patients who were diagnosed with neurofibromatosis type 1 (NF1), out of which 81 (50.62%) were males vs 79 (49.37%) were females. The mean age of the patients was 8.08 years (±5.21). The mean BMI was estimated to be 18.90 (±6.54).

Table 1 shows NF1 criteria components. Among our 160 patients, we found that the most evident clinical manifestation among the NF1 criteria is the presence of cutaneous café au lait spots or macules. Café au lait macules were observed in 133 (83.12%) patients. Moreover, neurofibromas, including the cutaneous and plexiform types, were also found among our patients. Cutaneous neurofibromas were expressed by 33 (20.62%) patients while 31 (19.37%) patients had plexiform neurofibromas. Also, iris Lisch nodules were reported in some patients. In this study, we found that 54 (33.75%) patients suffered from iris Lisch nodules and they were following up with an ophthalmologist. In addition to iris Lisch nodules, optic pathway glioma constitutes a part of the NF1 diagnostic criteria. We found that 29 (18.12%) patients had a reported history of optic pathway glioma. Moreover, axillary or inguinal skinfold freckling was observed in 44 (27.50%) patients. Skeletal abnormalities are also one of the NF1 criteria, as we found that 27 (16.87%) patients had skeletal involvement of the disease upon the diagnosis of NF1. Most importantly, 83 (51.87%) patients had at least one first-degree relative with NF1.

**Table 1:** Neurofibromatosis type 1 (NF1) diagnostic criteria

NF Criteria (N = 160)	Frequency (%)
Café au lait spots	133 (83.12)
Cutaneous neurofibroma	33 (20.62)
Plexiform neurofibroma	31 (19.37)
Iris Lisch nodules	54 (33.75)
Optic pathway glioma	29 (18.12)
Skinfold freckling	44 (27.50)
Skeletal Involvement	27 (16.87)
First-degree relative with NF1	83 (51.87)

Table 2 illustrates the past medical history of the included patients. In this study, we found that 35 (21.87%) patients out of 160 had a previously reported history of visual impairment. Also, 13 (8.12%) were reported to have complete visual loss, either unilateral or bilateral. Moreover, 27 (16.87%) patients were identified to have concurrent epilepsy requiring treatment with neurofibromatosis type 1. Additionally, a history of recurrent episodes of headache was noted among 27 (16.87%) patients. Also, some patients were suffering from short stature as was noticed in 24 (15%) patients. Furthermore, congenital bone deformities were observed in some patients, one of which is scoliosis. As 17 (10.62%) patients were identified to have scoliosis. Cognitive impairments and behavioral disorders were also evident among our patients. We found out that 15 (9.37%) patients were reported to have some degree of cognitive impairment. Moreover, in this study, we found that four (2.50%) patients, whose age was less than eight years, had a history of precocious puberty. Furthermore, 56 (35%) patients were identified to have brain tumors. Although, 85 (53.12%) had no significant past medical history.

**Table 2:** Associated medical history of patients with neurofibromatosis type 1

Associated medical history	Frequency (%)
Scoliosis	17 (10.62)
Epilepsy	27 (16.87)
Headache	27 (16.87)
Short stature	24 (15)
Visual impairment	35 (21.87)
Visual loss	13 (8.12)
Precocious puberty	4 (2.50)
Cognitive impairment	15 (9.37)
No significant medical history	85 (53.12)

**Figure 1:**
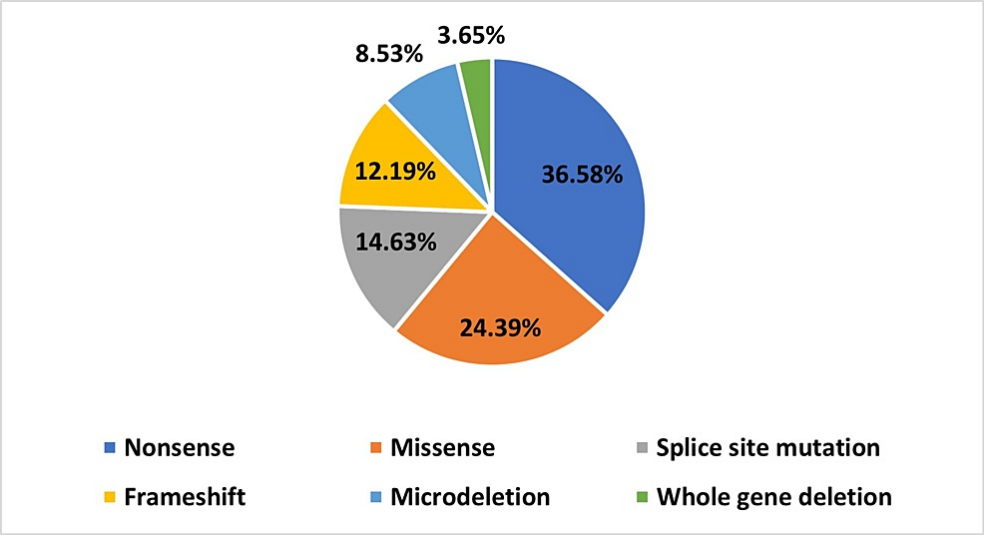
Types of mutations among patients with neurofibromatosis type 1

Table 3 demonstrates the genotype of our included patients. Among the 160 included patients, only 100 (62.50%) underwent NF1 genetic sequencing. Furthermore, 82 (82%) were identified to have a genetic mutation involving the NF1 gene while 18 (18%) had negative genetic testing. Out of the 82 patients who had positive genetic testing, heterozygosity was noted in 81 (98.78%) patients while only one patient (1.21%) had a homozygous mutation at the NF1 gene. As depicted in Figure 1, the nonsense type of mutation was the most common among our patients. As 30 (36.58%) patients were found to have a nonsense mutation upon NF1 genetic sequencing. Following nonsense mutations, missense mutation was evident among 20 (24.39%) patients. Also, 12 (14.63%) patients had splicing site mutation or defect. Frameshift mutation was observed in 10 (12.19%) patients. Moreover, seven (8.53%) had a microdeletion involving one or more of the NF1 exons. In addition, whole NF1 gene deletion was identified in three (3.65%) patients. Lastly, according to the findings of this study, we did not find any statistically significant correlation between the frequency of optic pathway glioma occurrence and specific types of mutations (p value= 0.626).

**Table 3:** Genetic-related parameters among patients with neurofibromatosis type 1

Genotype		Frequency (%)
Genetic testing (N=160)	Yes	100 (62.50)
	No	60 (37.50)
Results (N=100)	Positive	82 (82)
	Negative	18 (18)
Zygosity (N=82)	Heterozygous	81 (98.78)
	Homozygous	1 (1.21)
Type of genetic mutation	Nonsense	30 (36.58)
	Missense	20 (24.39)
	Splice site mutation	12 (14.63)
	Frameshift	10 (12.19)
	Microdeletion	7 (8.53)
	Whole gene deletion	3 (3.65)

## Discussion

This study looked at the clinical symptomology and neurogenetic features of NF1 in pediatrics at different tertiary care centers in Saudi Arabia. All patients in this research met the NF1 diagnostic criteria, with a mean of three criteria met per patient. Like previous studies, the presence of café-au-lait macules was the most prominent of the diagnostic criteria for NF1, as shown in 83.12% of our patients [18]. Iris Lisch nodules were seen in 33.75% of patients, which was consistent with the previous literature and was a significant indicator of the disease [18].

Likewise, 10.62% suffered from scoliosis, which was almost as high as the prior study's prevalence rate of 3% [18]. Epilepsy was the presenting feature in 16.87% of patients, almost double the previously reported incidence [18]. The clinical relevance of NF1 is highlighted by its ability to trigger the development of both benign and malignant tumors, typically in the nervous system; hence, NF1 ought to be detected early in children to identify said malignancies and promote health. Apart from the neurological system, NF1 has been linked to an elevated relative risk of other malignancies, such as neurofibromas, which represent benign nerve sheath tumors originating from non-myelinating Schwann cells, and they are considered a characteristic feature of NF1 [19]. Neurofibromas commonly manifest as cutaneous or subcutaneous nodules of varied sizes that appear anywhere on the body and are hardly seen in younger patients, as they frequently appear throughout puberty and tend to increase with age [20]. Plexiform neurofibromas typically include several nerve fascicles and are predominantly congenital [21]. Despite them being uncommon in the general population, they can progress to malignant peripheral nerve sheath tumors (MPNSTs) with a lifetime risk of 8-13% in individuals with NF1 [22]. Even though plexiform neurofibromas are thought to be pathognomonic for NF1, their incidence among our patients was considerably low when compared to other studies, likely due to the advanced mean age of our sample. On the other hand, cutaneous neurofibromas are not a concern regarding malignant transformation, yet patients frequently prefer these tumors to be excised because of the disfigurement and slight pain.

The most common type of brain tumor linked with NF1 is optic pathway glioma (OPG), which is often seen in children aged seven years and younger with an estimated prevalence rate of 15-20% [23]. The prevalence of OPG in our research was 18.12%, which was comparable to the literature. Nevertheless, OPGs in children are often inactive and asymptomatic and are frequently discovered incidentally during regular screening. Although some individuals may experience visual loss, severe proptosis, hydrocephalus, and early puberty [23]. Additionally, cerebral gliomas are a frequent finding in NF1. Even though most of them are low-grade, multiple cases of high-grade gliomas have been reported in the literature [24]. Brainstem gliomas are the second most prevalent brain tumors in NF1, accounting for 18% of all NF1 tumors in the central nervous system [25]. These tumors are less likely to be aggressive than sporadic occurrences of OPG and brainstem gliomas, thus some individuals remain asymptomatic. Malignancies of the posterior fossa are uncommon in NF1 and are anticipated to develop at a rate of 1% and 0.83%, respectively [26]. In our study, the prevalence rate of non-optic pathway gliomas was found to be 16.87%.

The co-occurrence of endocrine disorders in NF1 patients is common, including thyroid disorders, precocious puberty, and short stature, as they were investigated in a prior study [27]. According to our findings, short stature had the highest frequency of co-occurrence with NF1, as it was noted in 24 patients. Moreover, eight patients, all of whom were younger than eight years old, were reported to have precocious puberty.

Additionally, NF1 could affect cognitive functions in some patients at some point in their lives by provoking some changes in brain structures and networks, as a previous study has described that not only one but many domains of cognitive functions might be affected, which might give us a hint toward the fact that NF1 might result in a certain cognitive phenotype [28]. It was reported in the literature that the decline in cognitive function in patients with NF1 might result in an intelligence quotient (IQ) lower by one standard deviation when compared to the general population. Also, social cognition deficits and attention deficit and hyperactivity disorder (ADHD) have been highly linked to the phenotype of NF1 in pediatric patients [28]. The study on hands shows that only 15 patients were identified to have any kind of cognitive impairment, and ADHD was only observed in a couple of patients. It is important to identify said patients before the initiation of the treatment plan, as more attention and special care are needed. Also, a multidisciplinary team approach is very critical for those patients, as more frequent hospital visits might be required.

A study that analyzed the known NF1 mutations found that the most common form is a small deletion, with nucleotide substitution coming in second. Yet in our study, the most common form of mutation was nonsense, followed by missense, splicing site mutation, frameshift mutation, and then microdeletion. We found no phenotype-genotype correlation, and that could be attributed to many causes; the intricacy of the NF1 phenotype and the variety of pathogenic NF1 mutations are two examples. Moreover, it was described in the previous literature that splicing site mutations are highly linked to central nervous system gliomas and other CNS tumors [29]. However, in our study, only two patients were identified to have OPG and other tumors in the presence of a splicing site mutation.

As shown in Table 4, we compared important findings in this research to the previously reported percentages in the literature.

**Table 4:** Important features compared with other studies from the literature

Feature	Our study (%)	Other studies (%)	Reference
Optic pathway glioma	18.12	15-20	Melloni G et al [23]
Non-optic pathway glioma	16.87	4.4-5.2	Huson SM et al [2]
Scoliosis	10.6	3	Boulanger et al [18]
Epilepsy	16.87	3.2	Boulanger et al [18]
None sense mutation	36.58	27	Nahla N et al [30]
Splicing mutation	14.63	9	Nahla N et al [30]

Although there has been significant progress in understanding this disorder, various barriers remain. Managing patients with neurofibromatosis type 1 and developing successful therapies will need a coordinated and multidisciplinary approach. This study has some limitations, one of which is the retrospective chart review method being the only source of data collection. Also, we recommend conducting this study on a larger scale involving more tertiary hospitals to obtain the best overview.

## Conclusions

In conclusion, optic pathway glioma and other brain tumors were prevalent among Saudi patients with NF1 raising the importance of early detection of the disease to provide the optimal quality of life possible. NF1 might affect different aspects of the patient's health; hence, close monitoring of the disease is crucial to avoid preventive complications such as endocrine disorders and the development of cranial brain tumors. Given the various clinical presentations of NF1, a multidisciplinary team approach is of value to incorporate all the patient needs to provide the best quality of care.

## References

[1] Cimino PJ, Gutmann DH (2018). Neurofibromatosis type 1. Handb Clin Neurol.

[2] Huson SM, Harper PS, Compston DA (1988). Von Recklinghausen neurofibromatosis. A clinical and population study in south-east Wales. Brain.

[3] Hirbe AC, Gutmann DH (2014). Neurofibromatosis type 1: a multidisciplinary approach to care. Lancet Neurol.

[4] Wallace MR, Marchuk DA, Andersen LB (1990). Type 1 neurofibromatosis gene: identification of a large transcript disrupted in three NF1 patients. Science.

[5] Rouleau GA, Merel P, Lutchman M (1993). Alteration in a new gene encoding a putative membrane-organizing protein causes neuro-fibromatosis type 2. Nature.

[6] Hulsebos TJ, Plomp AS, Wolterman RA, Robanus-Maandag EC, Baas F, Wesseling P (2007). Germline mutation of INI1/SMARCB1 in familial schwannomatosis. Am J Hum Genet.

[7] Kallionpää RA, Uusitalo E, Leppävirta J, Pöyhönen M, Peltonen S, Peltonen J (2018). Prevalence of neurofibromatosis type 1 in the Finnish population. Genet Med.

[8] Gutmann DH, Ferner RE, Listernick RH, Korf BR, Wolters PL, Johnson KJ (2017). Neurofibromatosis type 1. Nat Rev Dis Primers.

[9] Miraglia E, Moliterni E, Iacovino C, Roberti V, Laghi A, Moramarco A, Giustini S (2020). Cutaneous manifestations in neurofibromatosis type 1. Clin Ter.

[10] Haddar S, Mahjoub A, Ben Abdesslem N, Ben Mrad S, Knani L, Mahjoub H (2020). Lisch nodules in neurofibromatosis type 1 [Article in French]. J Fr Ophtalmol.

[11] Delucia TA, Yohay K, Widmann RF (2011). Orthopaedic aspects of neurofibromatosis: update. Curr Opin Pediatr.

[12] Coelho SG, Loureiro MJ (2020). Neurofibromatosis-associated pulmonary hypertension: an ominous duo. BMJ Case Rep.

[13] Sellmer L, Farschtschi S, Marangoni M (2017). Non-optic glioma in adults and children with neurofibromatosis 1. Orphanet J Rare Dis.

[14] Friedrich RE, Nuding MA (2016). Optic pathway glioma and cerebral focal abnormal signal intensity in patients with neurofibromatosis type 1: characteristics, treatment choices and follow-up in 134 affected individuals and a brief review of the literature. Anticancer Res.

[15] Rony C, Aharoni S, Halevy A (2022). The utility of early brain MRI for patients with neurofibromatosis type 1 and optic pathway glioma: a long-term follow-up in a tertiary referral hospital. Neuropediatrics.

[16] Blanchard G, Lafforgue MP, Lion-François L (2016). Systematic MRI in NF1 children under six years of age for the diagnosis of optic pathway gliomas. Study and outcome of a French cohort. Eur J Paediatr Neurol.

[17] Santoro C, Picariello S, Palladino F (2020). Retrospective multicentric study on non-optic CNS tumors in children and adolescents with neurofibromatosis type 1. Cancers (Basel).

[18] Boulanger JM, Larbrisseau A (2005). Neurofibromatosis type 1 in a pediatric population: Ste-Justine's experience. Can J Neurol Sci.

[19] Karajannis MA, Ferner RE (2015). Neurofibromatosis-related tumors: emerging biology and therapies. Curr Opin Pediatr.

[20] Boyd KP, Korf BR, Theos A (2009). Neurofibromatosis type 1. J Am Acad Dermatol.

[21] Evans DG, Baser ME, McGaughran J, Sharif S, Howard E, Moran A (2002). Malignant peripheral nerve sheath tumours in neurofibromatosis 1. J Med Genet.

[22] Evans DG, Salvador H, Chang VY (2017). Cancer and central nervous system tumor surveillance in pediatric neurofibromatosis 1. Clin Cancer Res.

[23] Melloni G, Eoli M, Cesaretti C (2019). Risk of optic pathway glioma in neurofibromatosis type 1: no evidence of genotype-phenotype correlations in a large independent cohort. Cancers (Basel).

[24] Varan A, Şen H, Aydın B, Yalçın B, Kutluk T, Akyüz C (2016). Neurofibromatosis type 1 and malignancy in childhood. Clin Genet.

[25] Mahdi J, Shah AC, Sato A (2017). A multi-institutional study of brainstem gliomas in children with neurofibromatosis type 1. Neurology.

[26] Pascual-Castroviejo I, Pascual-Pascual SI, Viaño J, Carceller F, Gutierrez-Molina M, Morales C, Frutos-Martinez R (2010). Posterior fossa tumors in children with neurofibromatosis type 1 (NF1). Childs Nerv Syst.

[27] Alshahrani A, Abuoliat Z, Alshahrani AS, Al Balwi MA (2022). Prevalence of associated endocrine diseases in patients with neurofibromatosis type 1. Avicenna J Med.

[28] Baudou E, Nemmi F, Biotteau M, Maziero S, Peran P, Chaix Y (2019). Can the cognitive phenotype in neurofibromatosis type 1 (NF1) be explained by neuroimaging? A review. Front Neurol.

[29] Upadhyaya M (2010). Neurofibromatosis type 1: diagnosis and recent advances. Expert Opin Med Diagn.

[30] Abdel-Aziz NN, El-Kamah GY, Khairat RA, Mohamed HR, Gad YZ, El-Ghor AM, Amr KS (2021). Mutational spectrum of NF1 gene in 24 unrelated Egyptian families with neurofibromatosis type 1. Mol Genet Genomic Med.

